# Berberine Stabilizes the Arrhythmogenic Substrate in Obese Rats by Klotho-Mediated Attenuation of Oxidative Stress and Inflammation

**DOI:** 10.3390/ijms27135769

**Published:** 2026-06-26

**Authors:** Qinaer Beikan, Shuang Jiang, Suhua Qiu, Cong Li, Yanxing Han, Yuhong Wang, Jiandong Jiang

**Affiliations:** 1 State Key Laboratory of Bioactive Substances and Function of Natural Medicine, Institute of Materia Medica, Chinese Academy of Medical Sciences & Peking Union Medical College, 1 Xian Nong Tan Street, Beijing 100050, China; qinaer@imm.ac.cn (Q.B.); jiangshuang013970@renfu.com (S.J.); congl_0104@163.com (C.L.); hangyanxing@imm.ac.cn (Y.H.); jiangjd@imm.ac.cn (J.J.); 2 The Key Laboratory of New Drug Pharmacology and Toxicology, Department of Pharmacology, Hebei Medical University, 361 East Zhongshan Road, Shijiazhuang 050017, China; qiusuhua111@hotmail.com; 3 Beijing Key Laboratory of Key Technologies for Preclinical Research and Development of Innovative Drugs in Pharmacokinetics and Pharmacokinetics, Institute of Materia Medica, Chinese Academy of Medical Sciences & Peking Union Medical College, 1 Xian Nong Tan Street, Beijing 100050, China

**Keywords:** obesity, arrhythmias, berberine, klotho, oxidative stress, cardiac remodeling

## Abstract

Obesity increases susceptibility to ventricular arrhythmias due to an arrhythmogenic substrate by promoting oxidative stress and inflammation-driven cardiac remodeling. Klotho, an anti-aging protein that is reduced in obesity-related cardiovascular disease, protects against oxidative injury and inflammation. Berberine (BBR) has been demonstrated to have antiarrhythmic properties, but Klotho mediates these effects in obesity remains unclear. Here, high-fat diet (HFD)-induced obese rats were treated with BBR for 8 weeks. Surface electrocardiography showed BBR shortened prolonged QT, QTc, and Tp-Te intervals. Optical mapping of isolated hearts revealed that BBR eliminated arrhythmia susceptibility (60% to 0%) and stabilized cardiac electrophysiology by shortening action potential duration (APD_50_/APD_90_), reducing repolarization dispersion, normalizing conduction velocity, and improving abnormal intracellular Ca^2+^ handling. BBR also attenuated cardiac hypertrophy and fibrosis and increased expression of the potassium channel subunits *Kv4.2*, *Kv4.3*, and *KChIP2*. Furthermore, BBR suppressed oxidative stress and inflammation while upregulating circulating and tissue Klotho levels in obese rats. In ox-LDL-treated H9C2 cells, Klotho silencing abolished the antioxidative and anti-inflammatory effects of BBR, indicating that Klotho is required for its cardioprotective actions. These findings demonstrate that BBR stabilizes the arrhythmogenic substrate in obesity-related cardiac remodeling, at least partly through upregulation of Klotho expression and subsequent attenuation of oxidative stress and inflammation.

## 1. Introduction

The increasing prevalence of obesity has become a major global health concern and is associated with an elevated risk of ventricular arrhythmias and sudden cardiac death [1,2]. Accumulating evidence indicates that obesity promotes the development of an arrhythmogenic substrate, defined as structural, electrical, and molecular alterations in the myocardium that increase susceptibility to cardiac arrhythmias [3,4]. These alterations are driven largely by chronic oxidative stress, inflammation, and lipid overload, which collectively impair ion channel function, prolong myocardial repolarization, and increase repolarization heterogeneity [4,5]. Conventional antiarrhythmic therapies primarily target ion channels yet fail to address this complex pathological milieu, underscoring the need for mechanistically broader therapeutic strategies.

Metabolic stress in obesity induces excessive reactive oxygen species production and chronic low-grade inflammation, both of which are central to electrophysiological and structural cardiac remodeling [3,6,7]. Oxidative stress disrupts intracellular calcium homeostasis and downregulates potassium channel subunits, including Kv4.2, KChIP2 and Kv1.5, prolonging action potential duration and increasing dispersion of repolarization [8,9,10]. Inflammatory signaling further destabilizes electrophysiology by impairing ion channel activity and gap junction integrity [11], while sustained oxidative and inflammatory responses promote myocardial hypertrophy and fibrosis, reinforcing a self-perpetuating arrhythmogenic cycle [12,13,14]. Therapeutic strategies capable of simultaneously targeting these pathological processes may offer an effective approach for preventing ventricular arrhythmias. However, treatment options that address these interconnected mechanisms remain limited, highlighting the need for novel therapeutic interventions.

Klotho is a membrane-bound or soluble anti-aging protein that has emerged as an important regulator of metabolic and cardiovascular homeostasis [15]. Its protective activity is essential for the proper function of many organs. The kidney, as the primary source of circulating Klotho, produces the soluble form, which functions as an endocrine factor with systemic protective effects, including on the heart [16]. Klotho is also expressed in cardiac tissue, where it contributes to the maintenance of myocardial structural integrity and electrophysiological stability [17,18]. Reduced circulating Klotho levels have been associated with metabolic disorders, cardiovascular diseases, and increased oxidative injury [15,19]. Emerging evidence suggests that Klotho preserves myocardial homeostasis by attenuating oxidative stress and inflammation, as well as modulating ion channel function [20,21]. Whether Klotho participates in the regulation of obesity-associated arrhythmogenic substrate remains incompletely understood.

Berberine (BBR), a natural isoquinoline alkaloid widely used in traditional medicine, has demonstrated multiple beneficial effects in metabolic disorders and cardiovascular diseases. Previous studies have demonstrated that BBR exhibits multiple pharmacological properties, including antioxidant, anti-inflammatory, and lipid-lowering effects, as well as cardioprotective effects against cardiac arrhythmias and sudden death [22,23]. Our previous study demonstrates that BBR can upregulate cardiac Klotho expression and attenuate cardiac senescence [24]. Another study reports that BBR increases circulating Klotho levels and upregulates renal Klotho expression to protect against acute kidney injury [25]. In addition, BBR has been shown to modulate ion channel function and improve electrophysiological stability in diabetic rats [26].

Despite evidence that BBR exerts antiarrhythmic, antioxidant, and anti-inflammatory effects, whether these actions are mediated through Klotho signaling in the setting of obesity has not been investigated. Furthermore, the role of Klotho in obesity-associated arrhythmogenic remodeling remains poorly understood. Therefore, investigating Klotho as a potential mechanistic link between BBR treatment and stabilization of the obesity-related arrhythmogenic substrate may provide new insights into the mechanisms underlying obesity-associated ventricular arrhythmias. To address these knowledge gaps, the present study systematically evaluated the effects of BBR on ventricular arrhythmia susceptibility, cardiac electrophysiological remodeling, oxidative stress, and inflammation in HFD-induced obese rats and further examined the role of Klotho signaling using complementary in vivo and in vitro approaches.

## 2. Results

### 2.1. BBR Attenuates Obesity-Associated Arrhythmia Susceptibility in Rats

**Figure 1:**
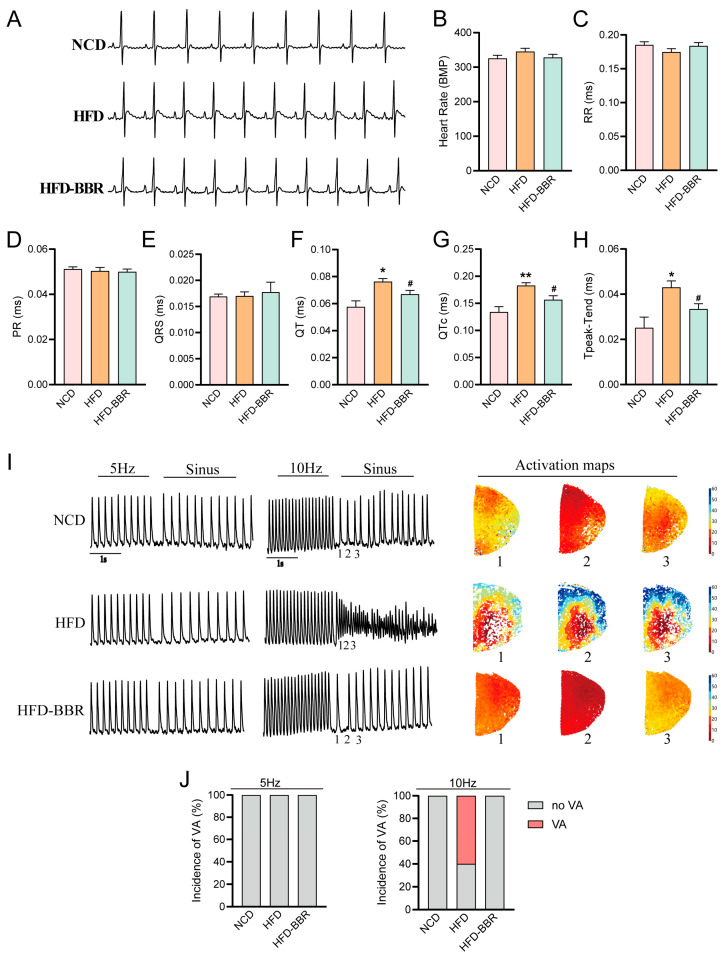
Effect of BBR on obesity-associated arrhythmia susceptibility in rats. (**A**) Representative ECG trace recordings from each group. (**B**–**H**) Quantitative analysis of ECG parameters, including heart rate (**B**), RR interval (**C**), PR interval (**D**), QRS duration (**E**), QT interval (**F**), QTc interval (**G**), and Tp-Te interval (**H**). (*n* = 8 per group) (**I**) Representative activation recordings, traces and maps obtained from ex vivo optical mapping. The time points labeled 1-3 on the traces correspond maps 1-3, respectively. (**J**) Quantitative analysis of ventricular arrhythmia (VA) incidence under programmed electrical stimulation (5 Hz and 10 Hz pacing) (*n* = 5 per group). Data are presented as mean ± SEM. * *p* < 0.05, ** *p* < 0.01 vs. NCD group; # *p* < 0.05 vs. HFD group. QTc, heart rate-corrected QT interval. BBR, berberine; ECG, electrocardiography; Tp-Te, Tpeak–Tend; NCD, normal chow diet; HFD, high-fat diet; HFD-BBR, high-fat diet with BBR treatment.

ECG recordings were performed to evaluate the effects of BBR on obesity-associated arrhythmia risk (Figure 1A–H). HFD-induced obesity significantly prolonged the QT interval, QTc interval, and Tp-Te intervals, indicating impaired ventricular repolarization, whereas BBR markedly shortened these intervals. The groups did not differ significantly in heart rate, RR interval, PR interval or QRS duration. These results suggest that BBR did not affect basal cardiac rhythm or atrioventricular conduction but improved obesity-associated repolarization abnormalities.

To evaluate arrhythmia inducibility under increased electrophysiological stress, Langendorff-perfused hearts were subjected to rapid pacing at 10 Hz using ex vivo optical mapping. Representative activation recordings, traces and maps are shown in Figure 1I. No ventricular arrhythmias were observed at baseline stimulation frequency (5 Hz) in any group (Figure 1I). At 10 Hz stimulation, no ventricular arrhythmias were observed in the NCD group; in contrast, arrhythmias characterized by irregular activation patterns were induced in three of five rats in the HFD group, while no arrhythmias were induced in the BBR-treated group (Figure 1J). These findings indicate that HFD impairs the electrophysiological adaptation of the heart to increased electrophysiological stress rather than baseline arrhythmias. The enhanced susceptibility at high-frequency pacing suggests reduced repolarization reserve and increased electrical instability in obese hearts. Collectively, these results demonstrate that BBR decreases arrhythmia inducibility in obese rats, reflecting an improvement in myocardial electrophysiological instability.

### 2.2. BBR Improves Cardiac Electrophysiological Instability in Obese Rats

**Figure 2:**
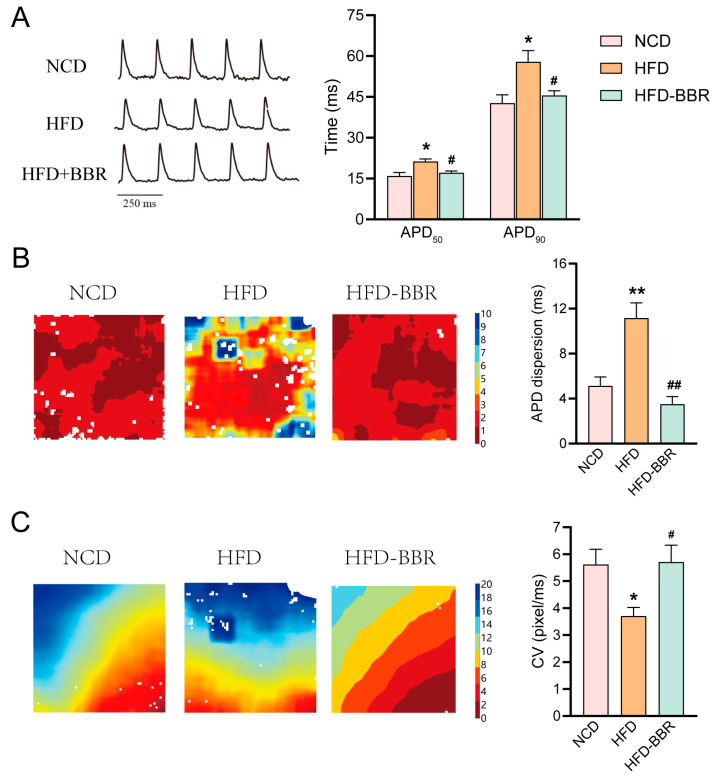
Effect of BBR on electrophysiological instability assessed by optical mapping in obese rat hearts. (**A**) Representative AP traces and quantitative analysis of APD_50_ and APD_90_ under 5 Hz pacing. (**B**) Representative heterogeneity in APD maps and quantitative analysis of dispersion of repolarization. (**C**) Representative activation maps and quantitative analysis of ventricular CV. Data are presented as mean ± SEM (*n* = 5 per group). * *p* < 0.05, ** *p* < 0.01 vs. NCD group; # *p* < 0.05, ## *p* < 0.01 vs. HFD group. BBR, berberine; APD, action potential duration; APD_50_, action potential duration at 50% repolarization; APD_90_, action potential duration at 90% repolarization; CV, conduction velocity; NCD, normal chow diet; HFD, high-fat diet; HFD-BBR, high-fat diet with BBR treatment.

Optical mapping was further performed to characterize the electrophysiological alterations underlying the antiarrhythmic effects of BBR. Under 5 Hz pacing, APD_50_ and APD_90_ were significantly prolonged in ventricles from HFD rats compared with NCD rats, indicating delayed ventricular repolarization (Figure 2A). BBR treatment significantly shortened both APD_50_ (*p* = 0.027) and APD_90_ (*p* = 0.037), suggesting shortened repolarization prolongation (Figure 2A). In addition, transmural dispersion of repolarization was markedly increased in HFD rats, as reflected by enhanced spatial heterogeneity of APD (Figure 2B). BBR significantly reduced this dispersion (*p* = 0.001), indicating improved electrical homogeneity of the ventricular myocardium (Figure 2B). Furthermore, ventricular CV, calculated from activation maps (Figure 2C), was significantly reduced in HFD rats compared with NCD rats, suggesting impaired electrical propagation. BBR treatment significantly increased CV (*p* = 0.043) (Figure 2C), indicating improved conduction properties. Collectively, these findings demonstrate that an arrhythmogenic substrate is formed in the obese heart and that BBR ameliorates electrophysiological instability, thereby exerting its antiarrhythmic effects.

### 2.3. BBR Improves Impaired Intracellular Ca^2+^ Handling in Obese Hearts

**Figure 3:**
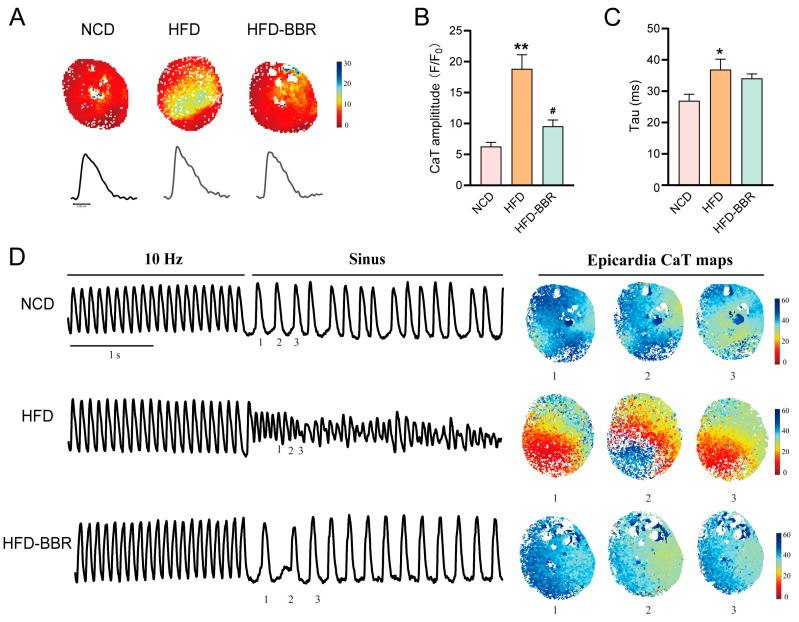
Effect of BBR on impaired intracellular Ca^2+^ handling in obese rats. (**A**) Representative intracellular CaT maps and corresponding CaT duration traces. (**B**) Quantitative analysis of CaT amplitude (F/F_0_). (**C**) Quantitative analysis of its decay time constant (Tau). (**D**) Representative epicardial CaT recordings, traces and maps obtained from ex vivo optical mapping at 10 Hz. The time points labeled 1-3 on the traces correspond maps 1-3, respectively. Data are presented as mean ± SEM (*n* = 5 per group). * *p* < 0.05, ** *p* < 0.01 vs. NCD; # *p* < 0.05 vs. HFD. BBR, berberine; CaT, calcium transient; NCD, normal chow diet; HFD, high-fat diet; HFD-BBR, high-fat diet with BBR treatment. Tau, the delayed time constant of calcium transient.

To further investigate the electrophysiological mechanisms underlying obesity-associated arrhythmia, ex vivo optical mapping was performed to assess calcium transient (CaT) dynamics. Representative intracellular CaT maps and corresponding CaT duration traces recorded at 5 Hz pacing are shown in Figure 3A. Quantitative analysis demonstrated that HFD significantly increased CaT amplitude and prolonged CaT decay constant (Tau) compared with the NCD group (Figure 3B,C), suggesting impaired intracellular Ca^2+^ handling in obese hearts. BBR treatment normalized Ca^2+^ transient amplitude (*p* = 0.003), suggesting improved intracellular Ca^2+^ handling, but did not affect Tau (Figure 3B,C). Under electrophysiological stress using rapid pacing at 10 Hz, markedly disorganized calcium waves emerged in the HFD group, reflecting a state of calcium dysregulation (Figure 3D), but not in the NCD and HFD-BBR groups. These findings indicate that BBR ameliorates excitation–contraction coupling abnormalities associated with obesity-induced electrical instability.

### 2.4. BBR Ameliorates Cardiac Structural and Ion Channel Remodeling

**Figure 4:**
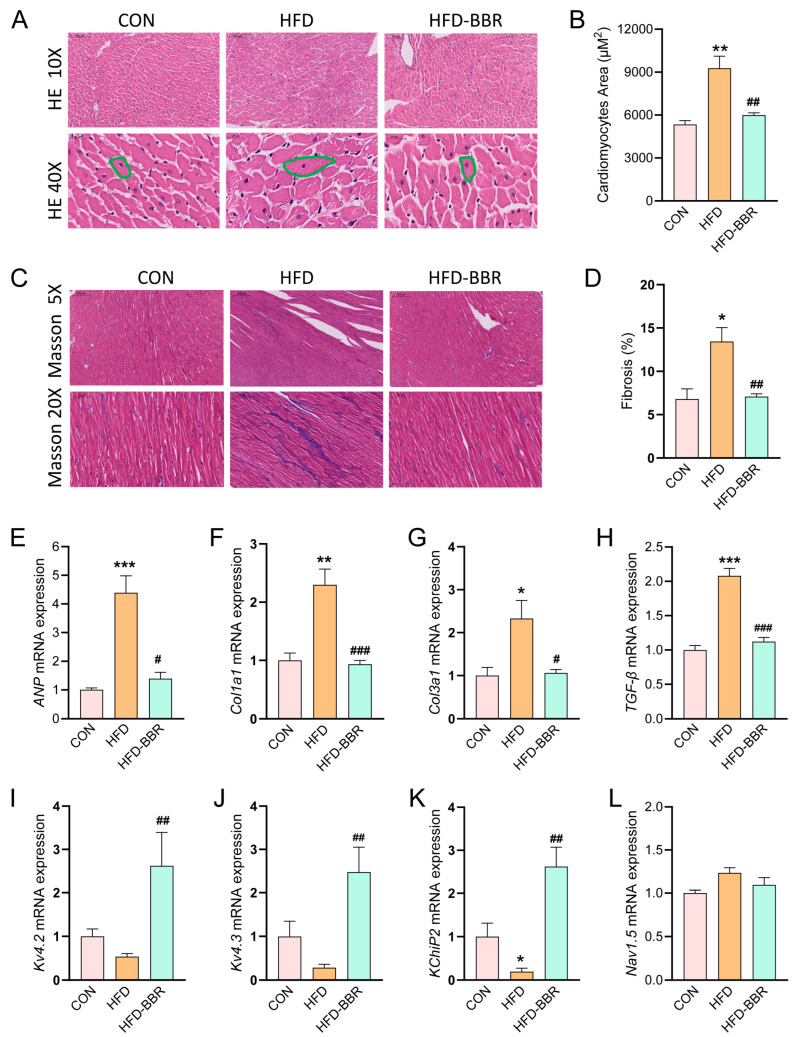
Effect of BBR on cardiac structural and electrical remodeling in obese rats. (**A**) Representative images of H&E staining (upper, 10×, scale bar = 100 µm; lower, 40×, scale bar = 20 µm). Green outlines indicate representative single cardiomyocyte used for cross-sectional area analysis. (**B**) Quantitative analysis of cardiomyocyte cross-sectional area. (**C**) Representative images of Masson staining (upper, 5×, scale bar = 200 µm; lower, 20×, scale bar = 50 µm). ((*n* = 3 per group)). (**D**) Quantitative analysis of cardiomyocyte cross-sectional area and fibrosis area. (**E**) Relative mRNA expression of the hypertrophic marker *ANP*. (**F**–**H**) Relative mRNA expression levels of fibrosis-related genes, including *Col1a1*, *Col3a1*, and *TGF-β*. (**I**–**K**) Relative mRNA expression levels of potassium channel subunits *Kv4.2*, *Kv4.3*, and *KChIP2*. (**L**) Relative mRNA expression level of sodium channel Nav1.5. Data are presented as mean ± SEM (*n* = 5 per group). * *p* < 0.05, ** *p* < 0.01, *** *p* < 0.001 vs. NCD group; # *p* < 0.05, ## *p* < 0.01, ### *p* < 0.001 vs. HFD group. BBR, berberine; H&E, hematoxylin and eosin; *ANP*, atrial natriuretic peptide; *TGF-β*, transforming growth factor-β; *KChIP2*, Kv channel-interacting protein 2; *Nav1.5*, sodium voltage-gated channel alpha subunit 5; NCD, normal chow diet; HFD, high-fat diet; HFD-BBR, high-fat diet with BBR treatment.

Cardiac remodeling contributes to the formation of an arrhythmogenic substrate that promotes the initiation and maintenance of cardiac arrhythmias. Compared with the control group, H&E staining revealed mild but discernible myocardial structural remodeling in HFD rats, characterized by enlarged cardiomyocytes without obvious myocardial necrosis or severe tissue disorganization (Figure 4A). Quantitative assessment demonstrated a significant increase in cardiomyocyte cross-sectional area (CSA), confirming the development of myocardial hypertrophy (Figure 4B). Masson staining showed increased collagen deposition, mainly in the interstitial and perivascular regions of the myocardium (Figure 4C). Quantitative analysis further demonstrated a significant increase in fibrotic area in HFD rats compared with controls (Figure 4D). These findings are consistent with the early-stage pathological characteristics of obesity-associated cardiac remodeling, which are primarily manifested by cardiomyocyte hypertrophy and progressive myocardial fibrosis. Consistently, mRNA expression of the hypertrophic marker *ANP* was significantly increased in HFD rats compared with NCD rats (Figure 4E). In addition, the expression of fibrosis-related genes, including *Col1a1*, *Col3a1*, and *TGF-β*, was markedly elevated (Figure 4F–H), further supporting the presence of obesity-induced myocardial hypertrophy and fibrotic remodeling. BBR treatment significantly attenuated myocardial hypertrophy and fibrosis and reduced the expression of *ANP*, *Col1a1*, *Col3a1*, and *TGF-β*, indicating an attenuation of obesity-associated cardiac remodeling.

To further investigate obesity-induced electrical remodeling, the expression of key ion channel genes involved in cardiac excitability and impulse conduction was examined. The mRNA expression levels of *Kv4.2*, *Kv4.3*, and *KChIP2* were reduced in HFD rats compared with NCD rats (Figure 4I–K), indicating impaired repolarization reserve. BBR treatment markedly increased the expression of these ion channel components. In contrast, no significant difference in *Nav1.5* mRNA expression was observed among the groups (Figure 4L). Collectively, these findings suggest that BBR alleviates both structural and electrical remodeling, thereby improving the arrhythmogenic substrate associated with obesity.

### 2.5. BBR Reduces Oxidative Stress and Inflammation

**Figure 5:**
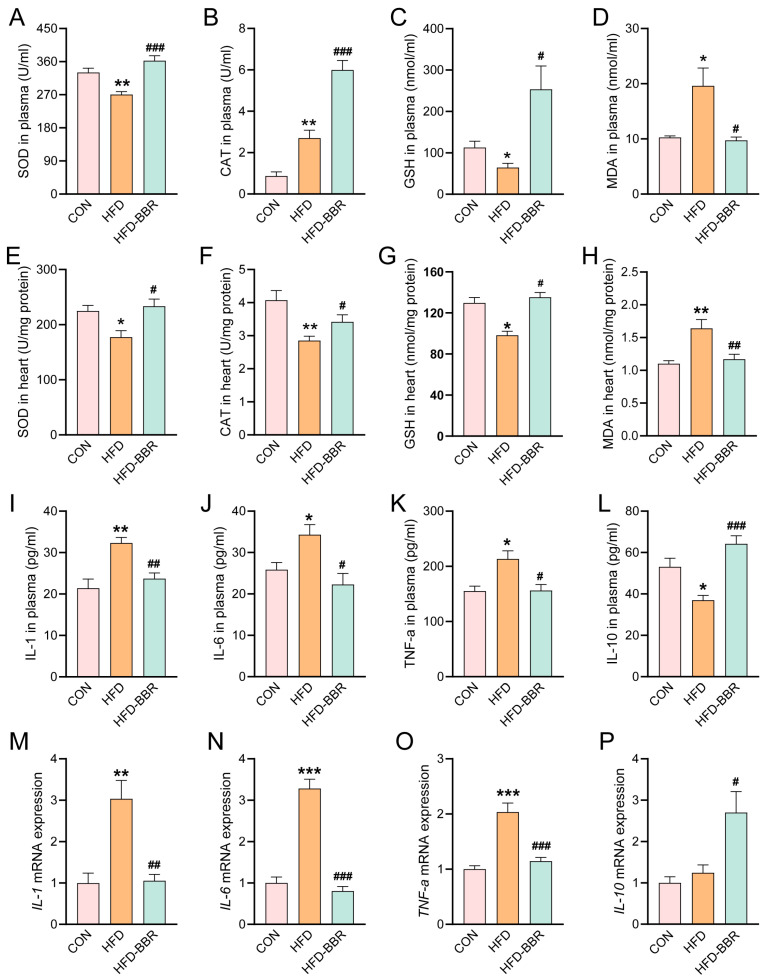
Effect of BBR on systemic and cardiac oxidative stress and inflammation in obese rats. Oxidative stress and inflammatory markers were evaluated in plasma and cardiac tissue, measured by ELISA or RT-PCR. (**A**–**D**) Activities of antioxidant enzymes SOD and CAT, and levels of GSH and MDA in plasma. (**E**–**H**) Activities of SOD and CAT, and levels of GSH and MDA in cardiac tissue. (**I**–**L**) Plasma concentrations of pro-inflammatory cytokines *IL-1*, *IL-6*, and *TNF-α*, and anti-inflammatory cytokine *IL-10*. (**M**–**P**) Relative mRNA expression levels of IL-1β, IL-6, TNF-α, and IL-10 in cardiac tissue. Data are presented as mean ± SEM (*n* ≥ 5 per group). * *p* < 0.05, ** *p* < 0.01, *** *p* < 0.001 vs. NCD group; # *p* < 0.05, ## *p* < 0.01, ### *p* < 0.001 vs. HFD group. BBR, berberine; SOD, superoxide dismutase; CAT, catalase; GSH, glutathione; MDA, malondialdehyde; *IL-1*, Interleukin-1; *IL-6*, Interleukin-6; *TNF-α*, tumor necrosis factor-α; *IL-10*, Interleukin-10; NCD, normal chow diet; HFD, high-fat diet; HFD-BBR, high-fat diet with BBR treatment.

Systemic and cardiac oxidative stress and inflammation are key contributors to obesity-associated cardiac remodeling. To assess the effects of BBR on redox homeostasis, oxidative stress markers were measured in both plasma and cardiac tissue (Figure 5A–H). Compared with NCD rats, HFD rats exhibited significantly decreased SOD activity and GSH levels, accompanied by a marked elevation in MDA levels, in both plasma and cardiac tissue; BBR treatment significantly reversed these alterations. In addition, plasma CAT activity was elevated in HFD rats, and BBR further augmented this increase; but in cardiac tissue, CAT activity was reduced in HFD rats, and BBR partially restored this reduction.

To further characterize the systemic inflammatory response, plasma levels of inflammatory cytokines were quantified (Figure 5I–P). Compared with NCD rats, HFD rats exhibited significantly increased levels of pro-inflammatory cytokines IL-1β, IL-6, and TNF-α, whereas the anti-inflammatory cytokine IL-10 was significantly decreased. BBR treatment markedly reversed these changes. Consistent with the plasma findings, quantitative PCR analysis of cardiac tissue revealed upregulated mRNA expression of *IL-1β*, *IL-6*, and *TNF-α* in HFD rats. BBR treatment not only attenuated the upregulation of pro-inflammatory cytokines but also significantly increased *IL-10* expression. These findings indicate that BBR attenuates obesity-associated systemic and cardiac oxidative stress and inflammation.

### 2.6. BBR Increases Circulating and Tissue Klotho Expression

**Figure 6:**
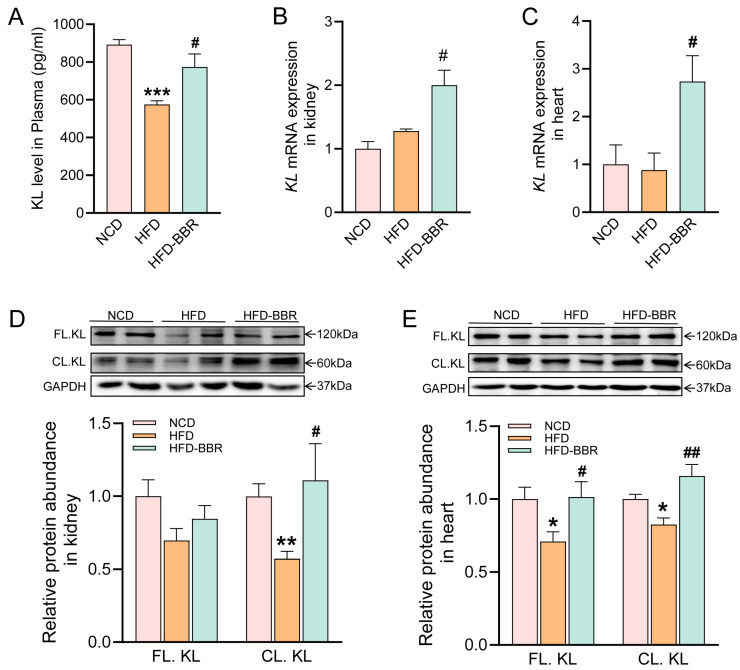
Effects of BBR on circulating and tissue Klotho expression in obese rats. Klotho expression levels were evaluated in plasma, kidney, and heart tissues. Circulating Klotho levels in plasma (**A**). Relative mRNA expression of *Klotho* in kidney tissue (**B**) and heart tissue (**C**). Representative Western blots showing protein levels of full-length membrane-bound Klotho (FL.Klotho, ~120 kDa) and cleaved soluble Klotho (CL.Klotho, ~60 kDa) in kidney tissue (**D**) and heart tissue (**E**). Data are presented as mean ± SEM (*n* ≥ 5 per group). * *p* < 0.05, ** *p* < 0.01, *** *p* < 0.001 vs. NCD group; # *p* < 0.05, ## *p* < 0.01 vs. HFD group. BBR, berberine; NCD, normal chow diet; HFD, high-fat diet; HFD-BBR, high-fat diet with BBR treatment.

As shown in Figure 6A, compared to NCD rats, circulating Klotho levels were significantly reduced in HFD rats, and BBR treatment significantly increased circulating Klotho levels (*p* = 0.001). In addition, BBR significantly increased both renal and cardiac *Klotho* mRNA expression levels in HFD rats (Figure 6B,C). Klotho protein exists in two major forms: the full-length membrane-bound form (FL.KL, ~120 kDa) and the cleaved soluble form (CL.KL, ~60 kDa). In Figure 6D,E, Western blot analysis further confirmed that Klotho protein expression in the kidney and heart was markedly reduced in HFD rats. BBR treatment significantly restored CL.KL protein expression in both cardiac (*p* = 0.001) and renal tissues (*p* = 0.043). However, BBR significantly restored FL.KL expression only in cardiac tissue; although FL-KL levels in renal tissue were decreased in HFD rats and showed an increasing trend following BBR treatment, the difference was not statistically significant.

### 2.7. Klotho Mediates the Protective Effects of BBR In Vitro

**Figure 7:**
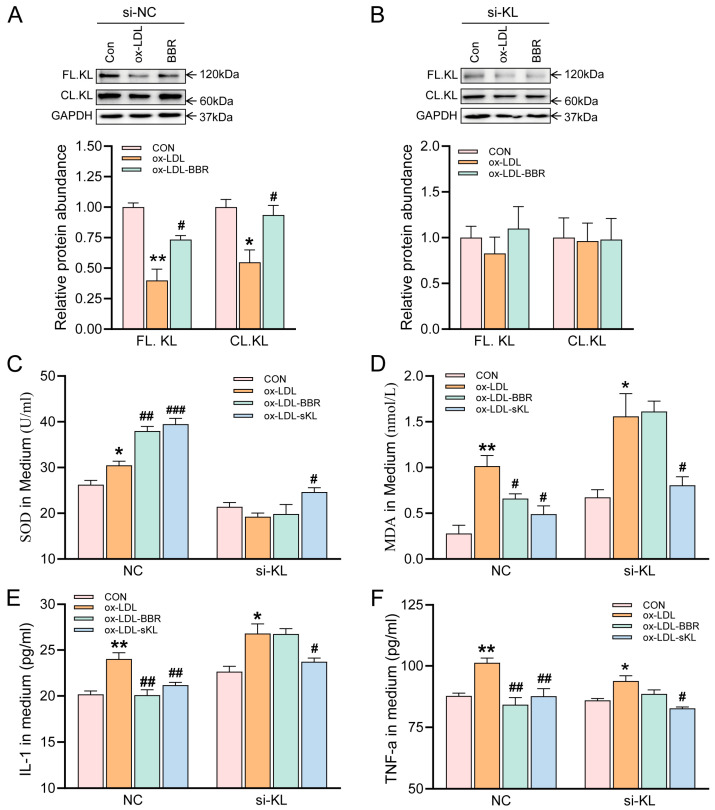
The role of Klotho in mediating the protective effects of BBR against ox-LDL-induced oxidative stress and inflammation in H9c2 cardiomyocytes. H9c2 cardiomyocytes were exposed to ox-LDL to mimic lipid-induced cellular injury. Cells were treated with BBR or recombinant soluble Klotho in the presence of control siRNA (siNC) (**A**) or Klotho-specific siRNA (siKL) (**B**). (**C**) Quantitative assessment of intracellular ROS activity. (**D**) Quantitative assessment of MDA levels. (**E**) IL-1 levels in the culture medium. (**F**) TNF-α levels in the culture medium. Data are presented as mean ± SEM (*n* = 3 independent experiments). * *p* < 0.05, ** *p* < 0.01 vs. control group; # *p* < 0.05, ## *p* < 0.01, ### *p* < 0.001 vs. ox-LDL group. BBR, berberine; SOD, superoxide dismutase; MDA, malondialdehyde; IL-1, Interleukin-1; TNF-α, tumor necrosis factor-α; NCD, normal chow diet; HFD, high-fat diet; HFD-BBR, high-fat diet with BBR treatment.

To investigate whether Klotho mediates the protective effects of BBR, H9c2 cardiomyocytes were exposed to oxidized low-density lipoprotein (ox-LDL) to mimic lipid-induced cellular injury. Ox-LDL treatment significantly reduced Klotho expression, whereas BBR treatment restored Klotho levels (Figure 7A). Knockdown of Klotho using siRNA abolished the BBR-induced upregulation of Klotho (Figure 7B). As shown in Figure 7C,E,F, ox-LDL exposure markedly increased oxidative stress and inflammatory responses in H9c2 cells, as evidenced by elevated MDA levels, and increased mRNA expression of pro-inflammatory cytokines including IL-1β and TNF-α. Treatment with either BBR or recombinant Klotho effectively reversed these effects. In addition, BBR or recombinant Klotho further increased SOD activity (Figure 7D). However, the effects of BBR were abolished following Klotho knockdown. These results indicate that Klotho is required for the antioxidant and anti-inflammatory effects of BBR and plays a critical role in mediating its cardioprotective actions.

## 3. Discussion

Obesity is increasingly recognized as an important risk factor for ventricular arrhythmias, largely due to the formation of an arrhythmogenic substrate characterized by structural and electrical remodeling, which induces oxidative stress and inflammation. In the present study, we demonstrated that BBR significantly stabilizes the arrhythmogenic substrate in obese rats, as evidenced by improved electrophysiological abnormalities, reduced cardiac hypertrophy and fibrosis, and decreased arrhythmia susceptibility. Importantly, our findings identify Klotho as a key mediator linking oxidative stress and inflammation to cardiac remodeling, providing new mechanistic insight into the antiarrhythmic effects of BBR in obesity.

Obesity is characterized by excessive lipid accumulation and mitochondrial dysfunction, leading to increased production of reactive oxygen species (ROS) and activation of inflammatory signaling pathways [27]. Oxidative stress disrupts ion channel expression and calcium homeostasis, leading to prolonged action potential duration and increased dispersion of repolarization, both of which are well-established determinants of arrhythmogenic substrate formation [6,7]. Consistent with previous studies, obese rats in the present study exhibited prolonged QT interval, increased action potential duration, slowed conduction velocity, and enhanced repolarization heterogeneity. These electrophysiological abnormalities indicate impaired electrical stability, thereby increasing susceptibility to ventricular arrhythmias. BBR treatment significantly improved these abnormalities, suggesting that BBR effectively stabilizes cardiac electrical activity under metabolic stress conditions.

Electrical remodeling is a central feature of the arrhythmogenic substrate. ROS and inflammatory mediators directly modify ion channel proteins through oxidative post-translational modifications and redox-sensitive signaling pathways (e.g., CaMKII, PKA), altering channel gating properties, reducing repolarization reserve, promoting early afterdepolarizations, and triggering arrhythmias [28,29]. Reduced expression of Kv4.2, Kv4.3, and KChIP2 has been associated with decreased transient outward potassium current (Ito), leading to delayed repolarization and increased arrhythmogenic risk [30]. In the present study, obesity significantly reduced expression of these potassium channel subunits, whereas BBR restored their expression, indicating improved repolarization reserve and electrical stability. Importantly, ion channel remodeling is not an isolated phenomenon but rather part of a broader pathological network involving oxidative stress and inflammation [31,32]. Therefore, interventions targeting upstream pathological processes may provide more effective protection against arrhythmias.

Abnormal intracellular Ca^2+^ handling is another key contributor to triggered arrhythmias. ROS-dependent activation of CaMKII has been shown to enhance RyR2 phosphorylation and spontaneous Ca^2+^ release, thereby promoting arrhythmogenic Ca^2+^ handling abnormalities in early-stage diabetes [33]. ROS-mediated oxidation of SERCA2a has been demonstrated to impair intracellular Ca^2+^ cycling and promote repolarization alternans in myocardial infarction [34]. Inflammatory signaling pathways may also interfere with mitochondrial function and intracellular calcium homeostasis, further exacerbating electrophysiological instability [35,36]. In HFD rats, we observed increased Ca^2+^ transient amplitude and prolonged Ca^2+^ decay kinetics, indicating abnormal Ca^2+^ handling. BBR treatment normalized Ca^2+^ transient amplitude, suggesting reduced sarcoplasmic reticulum (SR) Ca^2+^ release, possibly mediated by inhibition of RyR2 hyperphosphorylation or normalization of SR Ca^2+^ load. These findings indicate that BBR improves excitation–contraction coupling abnormalities associated with obesity-induced electrical instability. This restoration of Ca^2+^ handling, likely downstream of reduced oxidative stress, would be expected to suppress early and delayed afterdepolarizations (EADs/DADs) and thereby enhance electrophysiological stability.

Additionally, structural remodeling further contributes to arrhythmogenic substrate formation. Oxidative stress and inflammatory mediators can promote extracellular matrix remodeling and myocardial fibrosis, thereby increasing structural heterogeneity and conduction discontinuity [35,37]. In agreement with previous reports [38], obesity induced significant cardiac hypertrophy and fibrosis in our study, accompanied by increased expression of *ANP*, *Col1a1*, *Col3a1*, and *TGF-β*. BBR treatment significantly attenuated these structural alterations, indicating protective effects against obesity-associated myocardial remodeling, therefore reducing structural heterogeneity and improving electrical conduction uniformity.

Importantly, cardiac remodeling is not an isolated phenomenon but rather part of a broader pathological network involving oxidative stress and inflammation [31,32]. These processes interact synergistically, forming a mutually reinforcing vicious cycle that disrupts cellular homeostasis and promotes both electrical and structural remodeling [39]. Accumulating evidence suggests that cardiac arrhythmias arise not only from ion channel dysfunction but also from complex pathological substrates shaped by oxidative injury and chronic inflammation [40,41]. In the present study, BBR treatment alleviated systemic and cardiac oxidative stress and inflammation, consistent with its known antioxidant and anti-inflammatory properties in metabolic disorders [22,23]. Interestingly, plasma and myocardial CAT activities exhibited opposite responses in HFD-fed rats. Plasma CAT activity was elevated, whereas myocardial CAT activity was reduced. This differential pattern may reflect tissue-specific regulation of antioxidant defenses under obesity-associated oxidative stress. Elevated plasma CAT may represent a systemic compensatory response to increased circulating ROS, while persistent myocardial oxidative stress may overwhelm local antioxidant capacity, impairing CAT activity and heightening susceptibility to oxidative injury. BBR further enhanced plasma CAT while restoring myocardial CAT, suggesting its antioxidant effects encompass both reinforcement of systemic defenses and attenuation of local cardiac oxidative stress. Collectively, these findings indicate that BBR improves the proarrhythmic myocardial microenvironment by targeting upstream pathological processes, including oxidative stress and inflammation. Therefore, interventions aimed at these upstream mechanisms may provide more effective protection against obesity-associated arrhythmias than strategies focused solely on downstream ion channel abnormalities.

Reduced Klotho has been associated with metabolic disorders, increased oxidative stress, and cardiovascular dysfunction. Clinically, circulating Klotho levels are inversely associated with atrial fibrillation prevalence, supporting a protective role in electrophysiological stability [15,42,43]. Mechanistically, Klotho deficiency disrupts intracellular Ca^2+^ handling by increasing ryanodine receptor sensitivity and enhancing CaMKII-dependent phosphorylation, leading to abnormal Ca^2+^ release [44]. In addition, reduced Klotho expression has been shown to downregulate Kv4.2 expression and decrease Ito density, thereby prolonging ventricular repolarization duration and increasing arrhythmia susceptibility [45]. Restoration of Klotho expression normalizes Ca^2+^ handling and ion channel expression, stabilizing cardiac electrophysiological properties and attenuating ventricular arrhythmic events [17,44,45,46,47]. The antiarrhythmic effects are likely mediated in part by Klotho’s ability to inhibit NADPH oxidase activity and suppress NF-κB signaling, thereby reducing oxidative stress and inflammation [15]. In the present study, circulating and tissue Klotho levels were significantly decreased in obese rats, whereas BBR treatment markedly increased Klotho expression. In vitro experiments further demonstrated that Klotho silencing abolished the protective effects of BBR against ox-LDL-induced oxidative stress and inflammation, indicating that Klotho plays a critical role in mediating the protective effects of BBR. These findings position Klotho as a critical molecular bridge linking metabolic stress to electrical and structural remodeling.

Unlike conventional antiarrhythmic drugs that primarily modulate ion channel activity but often fail to correct the multifactorial pathological substrate responsible for electrical instability [48], increasing attention has therefore shifted toward therapeutic strategies targeting the arrhythmogenic substrate as an integrated network of oxidative stress, inflammation, and structural remodeling [49,50]. In this context, BBR exerted broad protective effects on myocardial homeostasis by significantly reducing oxidative stress and inflammatory responses while improving ion channel expression. Optical mapping further demonstrated that BBR shortened action potential duration, reduced repolarization dispersion, and improved conduction velocity, collectively indicating enhanced electrical stability. By restoring Klotho expression, BBR appears to interrupt the pathological cycle linking metabolic stress to electrophysiological dysfunction. Collectively, these findings suggest that BBR stabilizes the arrhythmogenic substrate in obesity through Klotho-mediated attenuation of oxidative stress and cardiac remodeling, highlighting the therapeutic potential of targeting the arrhythmogenic substrate in obesity-related ventricular arrhythmias.

Several limitations of this study should be acknowledged. First, ion channel remodeling was assessed only at the mRNA level, and protein expression or channel current measurements were not performed. Second, the molecular mechanisms underlying Ca^2+^ handling were not examined. Third, although our in vitro experiments support a mechanistic role for Klotho, further in vivo studies using genetic models are required to establish causality. Future studies are needed to validate these findings at the protein and electrophysiological levels, elucidate the role of Ca^2+^ handling in obesity-associated arrhythmogenesis, and further define the molecular mechanisms underlying Klotho-mediated cardioprotection. In addition, clinical studies will be necessary to evaluate the translational potential of BBR for the prevention and treatment of obesity-associated arrhythmias.

## 4. Materials and Methods

### 4.1. Animals and Experimental Design

To induce obesity, male 8-week-old Sprague Dawley rats (180–200 g) purchased from SPF Biotechnology Co. Ltd. (Beijing, China) were fed a HFD (1% cholesterol, 0.2% sodium cholate, 10% lard, 5% egg yolk powder, and 83.8% basal diet) supplied by Beijing Keao Xieli Feed Co., Ltd. (Beijing, China). After 8 weeks of HFD feeding, obesity was established, and the animals were randomly divided into three groups (*n* = 12 per group): (1) NCD group, (2) HFD group, and (3) HFD + BBR group. BBR (Macklin, Shanghai, China) or saline was administered by oral gavage for 8 weeks while the HFD was maintained, with the dose (100 mg/kg/day) based on our previous study [24].

All animals were housed in standard open-top polycarbonate cages (three rats per cage) with corncob bedding, under standard conditions (22–24 °C, 50–65% humidity, 12-h light/dark cycle), with free access to food and water. Rats were lightly anesthetized with 1.5% isoflurane in oxygen as needed. Euthanasia was performed by gradual carbon dioxide inhalation followed by confirmation of respiratory and cardiac arrest. All procedures were approved by the Institutional Animal Care and Use Committee of the Chinese Academy of Medical Sciences and followed the Guide for the Care and Use of Laboratory Animals and the ethical guidelines of the Chinese Council on Animal Care.

### 4.2. Electrocardiogram Recording and Analysis

As described previously [38], ECGs were recorded from rats lightly anesthetized with isoflurane (1.5% *v*/*v* in oxygen) one day prior to sacrifice. Recordings were obtained using a PowerLab acquisition system (ADInstruments, Castle Hill, Australia) after the animals had reached a stable physiological state for at least 5 min. Raw ECG data were exported and subsequently analyzed with LabChart version 8.0 software (ADInstruments). The following parameters were measured: heart rate, RR interval, QRS interval, QT interval, and Tpeak–Tend (Tp-Te) interval. The corrected QT interval (QTc) was calculated using Bazett’s formula: QTc = QT/(RR/100)1/2. All analyses were performed on ECG waveforms averaged from ten consecutive beats to minimize beat-to-beat variability.

### 4.3. Optical Mapping of Cardiac Electrophysiology

Optical mapping experiments were performed as previously described with minor modifications [38,51]. Briefly, rats were heparinized and anesthetized with 3% isoflurane, after which hearts were rapidly excised and mounted on a Langendorff perfusion system. Hearts were perfused with oxygenated Krebs solution (in mM: NaCl 119, NaHCO_3_ 25, KH_2_PO_4_ 1.2, KCl 4.0, MgCl_2_ 1.0, CaCl_2_ 1.8, glucose 10; pH 7.35–7.45; 95% O_2_/5% CO_2_). To minimize motion artifacts, blebbistatin (10 μM) was added to the circulating solution. The voltage-sensitive dye RH237 (1 μM) and calcium-sensitive dye Rhod-2 AM (5 μM) were loaded to record transmembrane voltage (Vm) and intracellular Ca^2+^ transients. Fluorescence signals were captured using an optical mapping system equipped with a high-resolution CCD camera (Little Joe, RedShirtImaging, Decatur, GA, USA). After reaching electrophysiological stability, optical mapping recordings were performed in sinus rhythm, and arrhythmias were induced by electrical stimulation with 5 Hz and 10 Hz with an isolated constant voltage/current stimulator (VCS3001, MappingLab Ltd., Oxford, UK). Optical signals were semi-automatically analyzed using OMapScope 5.0 software to quantify electrophysiological parameters, including action potential duration at 50% and 90% repolarization (APD50 and APD90), conduction velocity (CV), the amplitude of calcium transient (CaT) and the delay constant of CaT duration (Tau).

### 4.4. Histological Analysis

Left ventricular tissues from each group (*n* = 3 per group) were fixed in 4% paraformaldehyde for 24 h, dehydrated through graded ethanol, embedded in paraffin, sectioned at a thickness of 4 μm, and stained with H&E or Masson’s trichrome. Three non-consecutive sections per animal and five random non-overlapping fields per section were analyzed by a blinded investigator. Cardiomyocyte cross-sectional area (CSA) was quantified from H&E-stained sections by measuring transversely sectioned cardiomyocytes with centrally located nuclei. At least 100 cardiomyocytes were analyzed per animal. Myocardial fibrosis was quantified from Masson’s trichrome-stained sections as collagen volume fraction, calculated as the percentage of collagen-positive area relative to the total myocardial tissue area. Quantitative analyses were performed using ImageJ software (version 1.53, NIH, Bethesda, MD, USA), and the average value for each animal was used for statistical analysis.

### 4.5. Cell Culture and Treatment

H9c2 rat cardiomyoblasts were obtained from the Bena culture collection (BNCC, Henan, China) and cultured in Dulbecco’s modified Eagle’s medium (DMEM; Gibco, Grand Island, NY, USA) supplemented with 10% fetal bovine serum and 1% penicillin/streptomycin at 37 °C in a humidified atmosphere containing 5% CO_2_. Cells were seeded and allowed to reach 70–80% confluence prior to treatment. Cells were treated with oxidized low-density lipoprotein (ox-LDL) in the presence or absence of BBR or recombinant Klotho protein for 24 h.

### 4.6. Small Interferin

siKlotho and scrambled control siRNA were from Invitrogen (Carlsbad, CA, USA). The siKlotho sequences were: sense, 5′-UGUUCUUCAUACUCUUUGGGU-3′; and antisense, 5′-CCAAAGAGUAUGAAGAACAAC-3′. Transient transfection was performed per the manufacturer’s protocol, and knockdown efficiency was confirmed by Western blot. After transfection, the cells were treated and harvested for further experiments. This refers to our previous paper [24].

### 4.7. Western Blot Analysis

Protein lysates from left ventricular tissues or H9c2 cells were extracted using RIPA lysis buffer supplemented with protease inhibitors and quantified using a Bicinchoninic Acid (BCA) Protein Assay Kit (Thermo Fisher Scientific, Waltham, MA, USA). Equal amounts of protein (30 μg per lane) were separated by SDS-PAGE and transferred onto PVDF membranes (Millipore, Billerica, MA, USA). Membranes were blocked with 5% skim milk in TBST for 1 h at room temperature and then incubated overnight at 4 °C with primary antibodies against Klotho (1:1000, Cat No. GTX17093, GeneTex, Irvine, CA, USA) and GAPDH (1:5000, Cat No. 10494-1-AP, Proteintech, Wuhan, China). After washing with TBST, membranes were incubated with HRP-conjugated secondary antibodies (1:5000, Beyotime Biotechnology, Shanghai, China) for 2 h at room temperature. Protein bands were visualized using enhanced chemiluminescence (ECL) reagents (Millipore, Billerica, MA, USA) and captured using a Gel View 6000Pro II imaging system (Biolight Biotechnology, Guangzhou, China). Band intensities were quantified using ImageJ software (Version 1.53, National Institutes of Health, Bethesda, MD, USA) and normalized to GAPDH.

### 4.8. Quantitative Real-Time PCR

Total RNA from left ventricular tissue was extracted using the RNeasy Mini Kit (QIAGEN, Venlo, The Netherlands). RNA purity was assessed by A260/A280 ratio (1.8–2.0). A total of 1 μg of RNA was reverse-transcribed into cDNA using the HiFiScript Kit (CoWin Biotech, Taizhou, China). qPCR was performed using an UltraSYBR mixture on an ABI 7500 Fast system (Applied Biosystems, Waltham, MA, USA) under the following conditions: initial denaturation at 95 °C for 10 min, followed by 40 cycles of 95 °C for 15 s and 60 °C for 1 min. A melt curve was generated after each run to confirm amplicon specificity. Relative expression levels were normalized to GAPDH and calculated using the 2^−ΔΔCt^ method [52]. The primer sequences used in this study are listed in Table S1.

### 4.9. Biochemical Assays

Serum, tissue, and cellular lysates were analyzed for biochemical parameters. The levels of malondialdehyde (MDA) and glutathione (GSH) and activities of superoxide dismutase (SOD) and catalase (CAT) were assayed using commercial kits (Beyotime Biotechnology, Shanghai, China) per the manufacturer’s protocols, which are based on standard colorimetric methods for MDA (TBA reaction), GSH (DTNB method), SOD (NBT inhibition), and CAT (H_2_O_2_ decomposition). Inflammatory cytokines, including TNF-α, IL-1β, IL-6, and IL-10, were quantified using commercial sandwich ELISA kits (Mlbio, Shanghai, China) according to the manufacturer’s instructions.

### 4.10. Statistical Analysis

Statistical data are presented as mean ± SEM. Normality of the data was assessed using the Shapiro–Wilk test. All possible pairwise comparisons among groups were considered and performed using one-way analysis of variance (ANOVA) followed by Tukey’s post hoc tests for normally distributed data, or the Kruskal–Wallis test followed by Dunn’s multiple comparisons test for non-normally distributed data. Categorical arrhythmia incidence data were analyzed using Fisher’s exact test. All statistical analyses were carried out using GraphPad Prism 8.0 (GraphPad Software, San Diego, CA, USA). A *p*-value < 0.05 was considered statistically significant.

## 5. Conclusions

In conclusion, this study demonstrates that BBR stabilizes the arrhythmogenic substrate in obesity through a Klotho-dependent mechanism involving suppression of oxidative stress and cardiac remodeling. It reveals a previously unrecognized role of Klotho in mediating the antiarrhythmic effects of BBR in obesity.

## Data Availability

The original contributions presented in this study are included in the article/Supplementary Materials. Further inquiries can be directed to the corresponding author.

## Supplementary Materials

The following supporting information can be downloaded at: https://www.mdpi.com/article/10.3390/ijms27135769/s1.

## Abbreviation

The following abbreviations are used in this manuscript:

BBR	berberine
QTc	QT interval
HFD	high-fat diet
NCD	normal chow diet
ECG	electrocardiography
Tp-Te	Tpeak–Tend
APD	action potential duration
APD50	action potential duration at 50% repolarization
APD90	action potential duration at 90% repolarization
CV	conduction velocity
H&E	hematoxylin and eosin
CSA	cardiomyocyte cross-sectional area
ox-LDL	oxidized low-density lipoprotein
MDA	malondialdehyde
SOD	superoxide dismutase
GSH	glutathione
CAT	catalase
ANOVA	one-way analysis of variance
ANP	atrial natriuretic peptide
collagen I	collagen type I
collagen III	collagen type III
TGF-β	transforming growth factor-β
KChIP2	Kv channel-interacting protein 2
Nav1.5	sodium voltage-gated channel alpha subunit 5
ROS	reactive oxygen species
Ito	transient outward potassium current
SR	sarcoplasmic reticulum
EADs/DADs	early/delayed after depolarizations
