# Supplementary material for: Berberine Stabilizes the Arrhythmogenic Substrate in Obese Rats by Klotho-Mediated Attenuation of Oxidative Stress and Inflammation
Source: Int J Mol Sci. 2026 Jun 26;27(13):5769. doi: 10.3390/ijms27135769 (PMC13362301; doi:10.3390/ijms27135769)
Supplement: Supplementary file 1 [file ijms-27-05769-s001.zip › ijms-4341537-supplementary.pdf]

Supplementary Material

Table S1. Primer Sequences of the detected genes

**Gene Name**	**Forward**	**Reverse**
Nppa	5′-AGCCGAGACAGCAAACATCA-3′	5′-AGGTGGTCTAGCAGGTTCTTG-3′
Col1a1	5′-GGAGAGAGCATGACCGATGG-3′	5′-AAGTTCCGGTGTGACTCGTG-3′
Col3a1	5′-AGGTCCAGGGATACGGGGTA-3′	5′-CAGGGAAACCCATGACACCA-3′
*Tgf*b*1*	5′-GTGGCTGAACCAAGGAGACG-3′	5′-AGGTGTTGAGCCCTTTCCAG-3′
Kcnd2	5′-GACAACACTGGGGTATGGCG-3′	5′-CGATCACAGGCACGGGTAG-3′
Kcnd3	5′-CTCCAATGCCTACCTGCACA-3′	5′-GGTGGAGGTTCGTACAGACA-3′
Kcnip2	5′-GCTACTTTTCTCTTCAATGCCTT-3′	5′-AAACCAGCCACAAAGTCCTCAA-3′
Scn5a	5′-CTGTCCACTCTGGATGTCCATC-3′	5′-CTCTGCCGTCATGTTGTAATG-3′
*Il1*b	5′-TGGCAACTGTCCCTGAACTC-3′	5′-AAGGGCTTGGAAGCAATCCTTA-3′
Tgfa	5′-ATGGGCTCCCTCTCATCAGT-3′	5′-GCTTGGTGGTTTGCTACGAC-3′
Il6	5′-CCACCCACAACAGACCAGTA-3′	5′-TCCAGGTAGAAACGGAACTCC-3′
Il10	5′-TTCCCTGGGAGAGAAGCTGA-3′	5′-GACACCTTTGTCTTGGAGCTTA-3′
Kl	5′-GACGAGGACTCTTCTATGTCGAC-3′	5′-GGTTCTCAGGTAAAGGAGGGAAG-3′
Gapdh	5′-GGCTGCCTTCTCTTGTGACA-3′	5′-TCCCGTTGATGACCAGCTTC-3′
